# Genetic Analysis and Construction of a Fingerprint for Licensed *Triadica sebifera* Cultivars Using SSR Markers

**DOI:** 10.3390/plants13131767

**Published:** 2024-06-26

**Authors:** Qi Zhou, Baiqiang Chen, Dongyue Jiang, Fei Zhuge, Yingang Li

**Affiliations:** Zhejiang Academy of Forestry, 399 Liuhe Road, Hangzhou 310023, China; qizhou36@hotmail.com (Q.Z.); 15386433633@163.com (B.C.); jdyzjforestry@163.com (D.J.); zhugefei135@163.com (F.Z.)

**Keywords:** *Triadica sebifera*, fingerprinting, genetic relationship, breeding

## Abstract

*Triadica sebifera* is an important landscaping tree species because of its colorful autumn leaves. In recent years, some cultivars have been bred and licensed, but it can be difficult to identify them from their morphological traits due to their similar phenotypes. To explore the genetic relationships and construct a fingerprint of the cultivars, the licensed *T. sebifera* cultivars were analyzed using SSR markers. A total of 179 alleles were identified among the 21 cultivars at 16 SSR loci, and these alleles exhibited a high level of genetic diversity (He = 0.86). The genetic variations mainly occurred among cultivars based on an analysis of molecular variance (AMOVA). According to phylogenetic analysis, principal coordinate analysis (PCoA), and Bayesian clustering analysis, the genetic relationships were independent of geographic distances, which may be mainly due to transplantations between regions. Some cultivars with different leaf colors showed obvious genetic differentiation and may be preliminary candidates for cross-breeding. Finally, the fingerprint for the licensed cultivars was constructed with two SSR markers. The results of this study can provide technical support for the application and legal protection of licensed *Triadica sebifera* cultivars.

## 1. Introduction

*Triadica sebifera* is a native tree species of China and is mainly distributed in areas south of the Qinling Mountains and Huaihe River [1]. The seeds have been used for candles, heating oils, and cocoa butter equivalents since the late 1980s [2,3,4]. The leaves and roots can be used for herbal medicines [5]. In addition, *T. sebifera* is also an important tree with colored leaves and is often used in landscaping. The colors of *T. sebifera* leaves in autumn are relatively diverse, such as purple, red, orange, and yellow. In recent years, ornamental *T. sebifera* breeding has been carried out, and some ornamental colored cultivars have been developed [6,7]. There are 21 licensed *T. sebifera* cultivars in China with different autumn colors and different leaf traits, and these cultivars are also valuable materials for breeding. In general, the parents and the genetic characteristics of the germplasm are very important for ensuring that the target material or gene is passed on through breeding [8]. However, *T. sebifera* cultivars have mainly been derived from natural crosses, and their genetic backgrounds and relationships are also unclear, which is not conducive to the selection of desirable parents for cross-breeding [9]. Moreover, some cultivars also have similar phenotypes due to their closely related origins, and it is difficult to distinguish them based on morphological traits with obvious seasonal variations. Hence, there are homonyms (different cultivars with the same name) and synonyms (the same cultivar with different names) [10]. In order to apply and protect these cultivars, several questions remain to be resolved, such as the following: What are the genetic relationships and variations among the different *T. sebifera* cultivars? How can they be precisely identified without seasonal or locational limitations?

DNA-based molecular markers, including random amplified polymorphic DNA (RAPD) [11], intersimple sequence repeat (ISSR) [12], simple sequence repeat (SSR) [13], and single nucleotide polymorphism (SNP) [14], have been used for genetic analysis over the past several decades. Notably, SSRs are more consistent than RAPD, more polymorphic than ISSRs, and more easily genotyped than SNPs, and have been used in the analysis of genetic relationships and in the genetic identification of plants [15,16,17]. DNA fingerprinting is a molecular approach for identifying different cultivars [18]. To date, a series of fingerprinting databases based on SSRs have been established for plant species such as *Ginkgo biloba* [19], *Ailanthus altissima* [20], *Asparagus officinalis* [21], and *Juglans regia* [22]. These databases are important for cultivar identification and protection of cultivar rights. To date, studies on *T. sebifera* have focused mainly on phylogenetic relationships, chemical components, and biological activity [2,23,24]. In terms of molecular markers, several SSR markers have been developed and applied in the genetic analysis of *T. sebifera* populations [1,25,26,27]. Hence, SSR markers may be an effective method to study the genetic relationship and fingerprinting of *T. sebifera* cultivars.

In this study, SSR markers were selected for the genetic analysis of *T. sebifera* cultivars. The aim of this study was not only to explore the genetic diversity and genetic differentiation of *T. sebifera* cultivars but also to construct molecular fingerprinting for the cultivars. The findings of this study can guide the selection of ideal parents for *T. sebifera* breeding and can provide a technical basis for the protection of cultivar rights.

## 2. Results

### 2.1. Genetic Diversity

A total of 179 alleles were detected among the 16 SSR loci, ranging from 7 to 19. The average expected heterozygosity (He) and Shannon’s information index (I) were 0.86 and 2.12, respectively (Table 1). The SSR08 locus had the highest He (0.96), I (2.85), and polymorphism information content (PIC = 0.94) values, which revealed more abundant genetic information than the other loci. There were fewer alleles detected at SSR03 (7) and SSR14 (8) than at the other loci (9~19), and the level of genetic diversity at these two loci was lower than that at the other loci. There was a high proportion of *T. sebifera* homozygotes at 16 SSR loci. The observed heterozygosity (Ho) at 12 pairs of SSR loci was 0, while the Ho at the other 4 loci was much lower than the He.

The genetic diversity of the *T. sebifera* cultivars differed among regions (Table 2). The number of cultivars varied among different regions, and genetic diversity was abundant in regions with a high number of cultivars. There were more cultivars from Jiangsu (8) than from the other areas (2~7), and these cultivars had the highest number of alleles (Na = 6.13), He (0.84), I (1.68), and allelic richness (AR = 3.15) values. There were only two cultivars from Hubei, and their genetic diversity was lower than that of the cultivars from the other areas.

### 2.2. Genetic Differentiation

Analysis of molecular variance (AMOVA) revealed significant genetic variation among regions, among cultivars and within cultivars (Table 3; *p* < 0.01). Most of the genetic variations were detected among cultivars (88%). There was only 4% and 8% genetic variation among regions and within cultivars, respectively.

**Figure 1:**
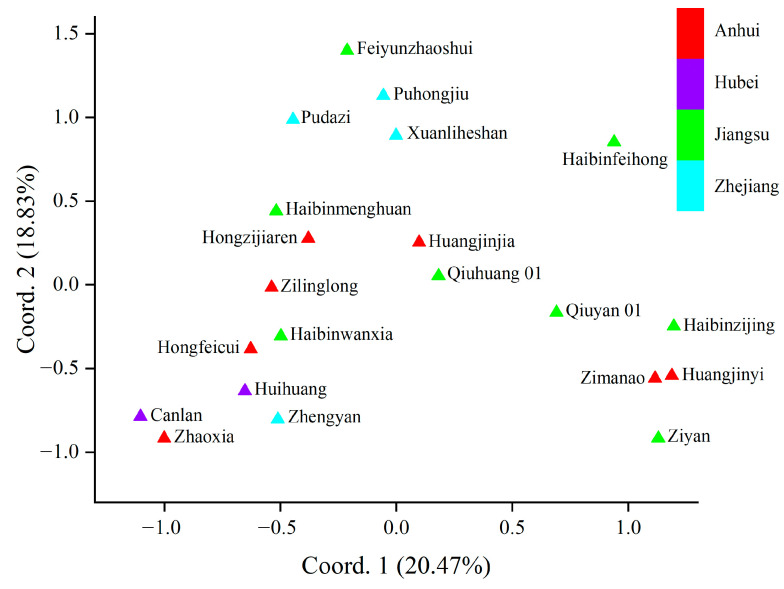
Results of principal coordinate analysis (PCoA).

**Figure 2:**
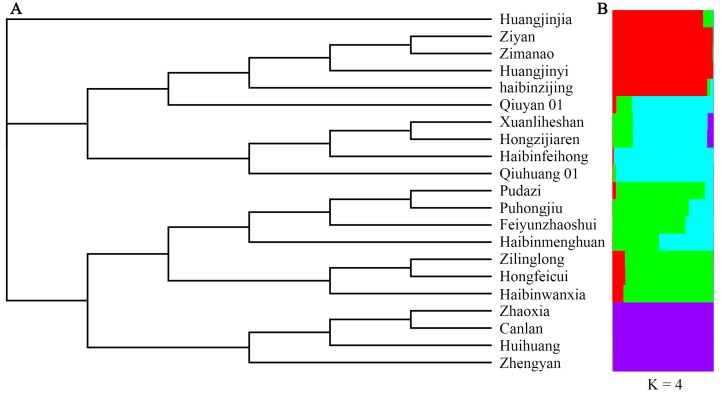
Phylogenetic clustering based on the neighbor-joining method (**A**) and Bayesian clustering at *K* = 4 (**B**).

To analyze the genetic relationships among the cultivars, principal coordinate analysis (PCoA) was carried out. The genetic relationships of the *T. sebifera* cultivars were independent of their origins (Figure 1). Only the cultivars from Hubei (Canlan and Huihuang) showed a close genetic relationship, while there were obvious genetic differences among the cultivars from other shared regions (Anhui, Jiangsu, or Zhejiang). Moreover, Bayesian clustering analysis and phylogenetic tree construction based on the neighbor-joining method were also carried out to analyze the genetic structure (Figure 2). The optimal *K* value was 4, which indicated that the cultivars were clustered into four groups (Figure S1). Most of the PCoA, Bayesian clustering analysis, and phylogenetic tree results were similar. However, according to the phylogenetic tree, the cultivar ‘Huangjinjia’ showed significant genetic variation and clustered into one group.

### 2.3. Unique Alleles

The frequencies of the 179 alleles at 16 SSR loci ranged from 0.0238 to 0.4524, and a total of 105 alleles were unique alleles, which were detected in only one cultivar (Table 4). Among the 16 SSR loci, there were 15 unique alleles at SSR08, while SSR03 and SSR14 had only 2 unique alleles. All the *T. sebifera* cultivars had unique alleles. The cultivar ‘Feiyunzhaoshui’ had the most unique alleles (10) at 10 SSR loci, the cultivars ‘Qiuyan 01’, ‘Qiuhuang 01’, and ‘Puhongjiu’ each had 8 unique alleles, and the cultivar ‘Zimanao’ had only one unique allele (156 bp at SSR12).

### 2.4. Fingerprinting of Cultivars

**Figure 3:**
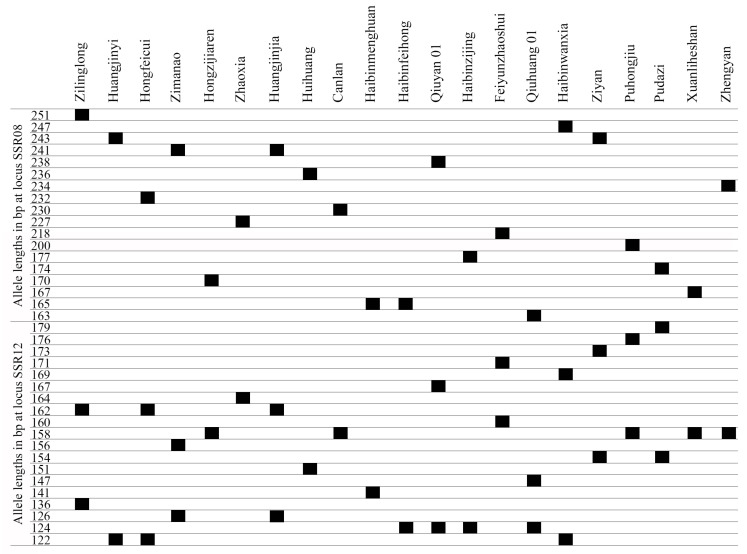
Fingerprints of the 21 cultivars based on 2 SSR loci (SSR08 and SSR12).

To distinguish and identify the *T. sebifera* cultivars, a fingerprint was constructed based on the alleles at 16 SSR loci. It was not possible to distinguish all the *T. sebifera* cultivars with only one SSR locus. The PIC of SSR08 and SSR12 were 0.94 and 0.90, respectively, which were higher than those of the other SSR loci. A total of 15 of the 21 *T. sebifera* cultivars were distinguished when neither SSR08 nor SSR12 was used. In total, the 21 *T. sebifera* cultivars were successfully distinguished by combining SSR08 and SSR12 (Figure 3).

## 3. Discussion

Genetic diversity contributes to the ability of a species to adapt to environmental changes and is valuable for evolution and conservation [28,29]. In plant breeding, genetic diversity can be described as the range of genetic characteristics and is an important resource for the development of new cultivars [30]. In this study, the 21 *T. sebifera* cultivars exhibited high genetic diversity (He = 0.86; I = 2.12). The genetic diversity of *T. sebifera* was greater than that of forest and fruit tree taxa such as *Populus* [31], *Olea europaea* [32], *Corylus avellana* [33], *Prunus avium* [34], and *Malus pumila* [35]. Moreover, there were significant differences in the phenotypic traits of the 21 *T. sebifera* cultivars, which may be a reflection of the high level of genetic diversity [36]. In general, natural populations may contain abundant genetic diversity [37]. The wild *Ipomoea batatas* individuals have a higher degree of genetic diversity than the cultivars [38]. However, the genetic diversity of widespread *T. sebifera* populations (He = 0.491) is much lower than that of *T. sebifera* cultivars [1]. The main reason may be that a wide range of *T. sebifera* trees have been destroyed or transplanted, which may lead to a loss of both germplasm and genetic diversity. Cross-breeding of plants, including natural and artificial hybrids, is an important approach for the generation of new cultivars. In plant breeding, large numbers of genetically stable and homozygous individuals are necessary, and complete homozygote breeding materials are rapidly obtained with doubled haploid technology, which is currently a routine part of breeding programs [39,40]. In this study, according to the 16 pairs of SSR markers, most of the cultivars had homozygous genotypes, which may be conducive to the inheritance of target genetic information in cross-breeding. In contrast, *Prunus armeniaca* [41] and *Prunus domestica* [9] cultivars have a high proportion of heterozygotes, and the Ho are 0.63 and 0.88, respectively. The main reason may be that *T. sebifera* cultivars have more inbreeding than *P. armeniaca* and *P. domestica* cultivars.

Some factors, such as geographic distance and selection, may influence genetic differentiation among populations [42]. Cultivars from different regions can also show genetic differentiation, and the cluster results of pear cultivars show a good fit with geographic distances [43]. However, the AMOVA in this study showed that most of the genetic variation was among cultivars (88%), and only 4% of the genetic variation was detected among regions. There were also limited genetic variations among regions in *Panax ginseng* and *Cajanus cajan* cultivars [44,45]. The main reason may be the gene flow between regions. The cultivars within the same region showed obvious genetic differentiation based on the PCoA, Bayesian clustering analysis, and phylogenetic tree results, indicating that the clustering result is independent of geographic distance. The *T. sebifera* cultivars from different regions also have similar phenotypes [36]. *T. sebifera*, an important economic tree species, has been widely distributed and transplanted across different regions over the last century due to its strong adaptability, which may be the reason for the low genetic differentiation among regions [1]. Genetic relationships between parents during cross-breeding may influence outcomes in genome-assisted breeding, and the close genetic distance between hybrid parents may cause hybrid disadvantages, including hybrid inactivation and hybrid decline [46,47]. Currently, *T. sebifera* is mainly used in landscaping because of its colorful leaves in autumn, and the main trait that is targeted in breeding is leaf color. Several cultivars with different leaf colors, such as ‘Puhongjiu’ (red), ‘Haibinfeihong’ (purple), ‘Huangjinjia’ (yellow), and ‘Zhaoxia’ (orange), exhibit great genetic differences and could be selected by strategic breeding of the parents. In contrast, complex genetic relationships may influence the study of the molecular regulation of leaf color via multiple omics analyses [48,49]. Therefore, cultivars with close genetic relationships, such as ‘Huihuang‘ (yellow) and ‘Zhengyan‘ (purple), may be good research materials for multiple omics analyses aimed at revealing the genetic basis of leaf color.

Given that *T. sebifera* cultivars are sometimes difficult to distinguish based on phenotypes, the inability to accurately identify the cultivars limits their applied use and the protection of cultivar rights. On the one hand, the main difference among *T. sebifera* cultivars is autumn leaf color, which is difficult to ascertain in other seasons when the leaves have not yet changed color. On the other hand, the phenotype of plants is controlled by both genetic and environmental factors, and a cultivar planted in different environments may have different morphological traits, which may cause misidentification and confusion [50]. When the *T. sebifera* cultivars were planted under the same conditions, some of them also had similar phenotypes [36]. Here, molecular fingerprinting is an accurate identification method without seasonal or locational limitations that relies on unique alleles and genotypes. In this study, there were 105 unique alleles identified at 16 SSR loci, which suggests that the genetic information among cultivars is noticeably different. In previous studies, fingerprinting of 10 *Trifolium repens* cultivars was performed using three representative SSR primers [10], while 24 *A. officinalis* cultivars were fingerprinted using three SSR primers [21]. However, the geographic origins of 110 rice cultivars cannot be fully identified only with SSR markers [51]. In the present study, just one SSR marker (SSR08 or SSR12) enabled approximately 70% of the cultivars (15) to be distinguished. Finally, the fingerprinting was constructed with only two SSR markers (SSR08 and SSR12). Hence, it can be speculated that the large quantity of polymorphic SSR markers and the high level of genetic differentiation among cultivars are important factors for the successful construction of a fingerprint with a small number of markers.

## 4. Materials and Methods

### 4.1. Plant Materials

**Figure 4:**
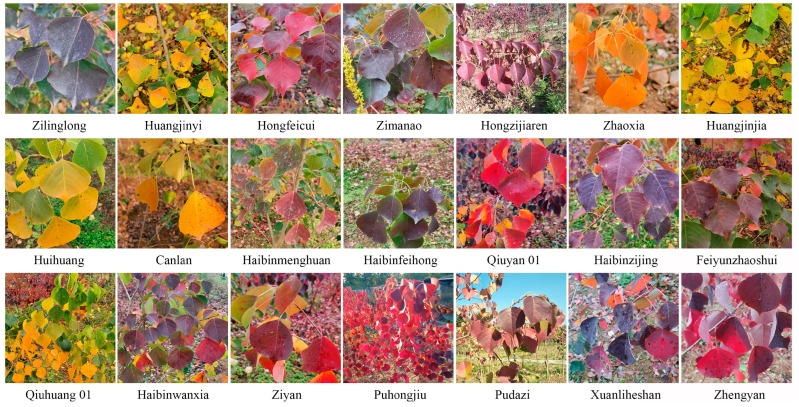
Differences in leaves among the 21 cultivars.

To date, a total of 21 *T. sebifera* cultivars have been licensed in China [36] (Table 5; Figure 4). The ‘Puhongjiu’ cultivar was approved by the Zhejiang Forestry Administration, while the other 20 cultivars were approved by the National Forestry and Grassland Administration. The scions of these cultivars were grafted onto two-year-old rootstocks, and the clones of these cultivars were cultivated in the *T. sebifera* germplasm nursery at Zhejiang Academy of Forestry Base (30°13′ N, 120°01′ E, 19 m above sea level). Young leaves of the 21 cultivars were collected and stored at −80 °C for later use.

### 4.2. DNA Extraction

Whole-genomic DNA was extracted using the DNeasy Plant Mini Kit (Qiagen, Hilden, NRW, Germany). The DNA was measured with a NanoDrop-1000 spectrophotometer (NanoDrop Technologies, Wilmington, DE, USA) and 1% agarose gel electrophoresis, and DNA with no significant degradation was used for polymerase chain reaction (PCR). Finally, the DNA was diluted to 60 ng/μL and stored at 4 °C for later use.

### 4.3. Genotyping with SSR Markers

A total of 39 SSR markers were selected from the references and tested via PCR amplification of six random individuals [1,25,27]. Sixteen of these SSR markers yielded clear and polymorphic products, and the upstream primers were fluorescently labeled with 5-Carboxyfluorescein (Table 6). The amplification of all samples at the 16 SSR loci was completed with an ABI Veriti 96 PCR system (Thermo Fisher Scientific, Waltham, MA, USA). The reaction mixtures (20 μL) contained 60 ng of DNA, 10 μL of 2 × TSINGKE Master Mix, and 0.2 μM of each SSR forward and reverse primer. The PCR program involved a predenaturation step of 4 min at 94 °C, followed by 30 cycles at 94 °C for 20 s, the appropriate annealing temperature for 30 s, and 72 °C for 90 s, and an extension step of 1 min at 72 °C. The PCR products of the amplifications were subjected to capillary electrophoresis using an ABI 3730xl instrument (Thermo Fisher Scientific, Waltham, MA, USA), and the fluorescent PCR bands were recognized. Finally, the genotype of each marker was analyzed using Peak Scanner v 1.0 (Thermo Fisher Scientific, Waltham, MA, USA) [52], and the base sizes of the bands were calculated with standard markers. The bands with the same base sizes were the same alleles in each pair of SSR markers. The alleles amplified in only one cultivar were unique alleles.

### 4.4. Data Analysis

The number of alleles (Na), effective number of alleles (Ne), observed heterozygosity (Ho), expected heterozygosity (He), Shannon’s information index (I), polymorphism information content (PIC), and phylogenetic analysis were calculated using PowerMarker version 3.25 (North Carolina State University, Raleigh, NC, USA) [53]. The allelic richness (AR) was measured with FSTAT version 2.9.3 (University of Lausanne, Lausanne, VD, Switzerland) [54]. Analysis of molecular variance (AMOVA) and principal coordinate analysis (PCoA) were performed with GenAlEx version 6 (Rutgers University, New Brunswick, NJ, USA) [55]. To study the genetic relationships among the cultivars, Bayesian clustering analysis was performed using Structure version 2.3.1 (Stanford University, San Francisco, CA, USA) [56].

## 5. Conclusions

The 21 licensed *T. sebifera* cultivars were analyzed using SSR markers, which revealed a high level of genetic diversity. The licensed cultivars showed homozygous genotypes at most SSR loci, which may be conducive to ensuring the inheritance of target genetic information in cross-breeding. Most of the genetic variation identified occurred among cultivars, and the genetic differentiation between them was independent of geographic distance, which may be mainly due to transplantations of this species across regions. *T. sebifera* cultivars with different leaf colors and obvious genetic differences were deemed to be important parents for cross-breeding. Finally, a fingerprint of the licensed *T. sebifera* cultivars was constructed with two markers.

## Figures and Tables

**Table 1:** Results of the genetic analysis of the 16 SSR loci.

Locus	Sample Size	Na	Ne	Ho	He	I	PIC
SSR01	21	11	6.78	0.00	0.87	2.15	0.84
SSR02	21	12	8.02	0.00	0.90	2.28	0.86
SSR03	21	7	3.97	0.00	0.77	1.64	0.72
SSR04	21	14	9.38	0.00	0.92	2.45	0.88
SSR05	21	10	7.00	0.00	0.88	2.11	0.84
SSR06	21	10	5.31	0.00	0.83	1.95	0.79
SSR07	21	10	4.41	0.24	0.79	1.81	0.75
SSR08	21	18	16.33	0.00	0.96	2.85	0.94
SSR09	21	11	6.58	0.00	0.87	2.14	0.83
SSR10	21	10	5.88	0.00	0.85	2.02	0.81
SSR11	21	9	5.58	0.00	0.84	1.95	0.80
SSR12	21	19	10.26	0.52	0.92	2.64	0.90
SSR13	21	10	7.00	0.00	0.88	2.14	0.84
SSR14	21	8	3.90	0.24	0.76	1.69	0.72
SSR15	21	10	6.63	0.14	0.87	2.08	0.83
SSR16	21	10	5.88	0.00	0.85	2.02	0.81
Mean	21	11.19	7.06	0.07	0.86	2.12	0.82

Na: number of alleles; Ne: effective number of alleles; Ho: observed heterozygosity; He: expected heterozygosity; I: Shannon’s information index; PIC: polymorphism information content.

**Table 2:** Genetic diversity of the cultivars from different regions.

Regions	Sample Size	Na	He	I	AR
Anhui	7	4.81	0.78	1.44	2.92
Hubei	2	1.75	0.50	0.52	1.75
Jiangsu	8	6.13	0.84	1.68	3.15
Zhejiang	4	3.56	0.73	1.15	2.72
Mean		4.06	0.71	1.20	2.63

AR: allelic richness.

**Table 3:** AMOVA of 21 cultivars from 4 regions.

Source of Variance	Variance Component	Percentage of Total	*p* Value
Among regions	0.27	4%	<0.01
Among cultivars	6.27	88%	
Within cultivars	0.57	8%	
Total	7.11	100%	

**Table 4:** The unique alleles of the 21 cultivars at different loci.

Cultivars	Unique Allele Lengths in bp (Locus)	Number of Unique Alleles
Zilinglong	413 (SSR01); 251 (SSR08); 192 (SSR09); 136 (SSR12); 157 (SSR13)	5
Huangjinyi	204 (SSR06); 215 (SSR15)	2
Hongfeicui	134 (SSR05); 232 (SSR08); 190 (SSR11)	3
Zimanao	156 (SSR12)	1
Hongzijiaren	238 (SSR07); 169 (SSR08)	2
Zhaoxia	391 (SSR01); 192 (SSR04); 163 (SSR05); 227 (SSR08); 196 (SSR09); 164 (SSR12); 200 (SSR15)	7
Huangjinjia	411 (SSR01); 225 (SSR06); 202 (SSR15)	3
Huihuang	286 (SSR02); 210 (SSR04); 262 (SSR06); 236 (SSR08); 166 (SSR10); 151 (SSR12)	6
Canlan	396 (SSR01); 298 (SSR02); 202 (SSR04); 170 (SSR05); 249 (SSR06); 230 (SSR08); 208 (SSR15)	7
Haibinmenghuan	119 (SSR03); 126 (SSR05); 159 (SSR10); 141 (SSR12); 162 (SSR16)	5
Haibinfeihong	409 (SSR01); 184 (SSR11); 193 (SSR15)	3
Qiuyan 01	402 (SSR01); 335 (SSR02); 185 (SSR04); 230 (SSR06); 188 (SRR07); 238 (SSR08); 205 (SSR09); 167 (SSR12)	8
Haibinzijing	280 (SSR02); 173 (SSR08); 157 (SSR10)	3
Feiyunzhaoshui	195 (SSR04); 241 (SSR06); 218 (SSR08); 194 (SSR09); 153 (SSR10); 167 (SSR11); 160 (SSR12); 170 (SSR13); 178 (SSR14); 177 (SSR16)	10
Qiuhuang 01	275 (SSR02); 228 (SSR06); 190 (SSR07); 163 (SSR08); 145 (SSR10); 187 (SSR11); 147 (SSR12); 150 (SSR16)	8
Haibinwanxia	290 (SSR02); 115 (SSR03); 187 (SSR04); 244 (SSR08); 208 (SSR09); 169 (SSR12); 164 (SSR16)	7
Ziyan	183 (SSR04); 173 (SSR12); 206 (SSR15)	3
Puhongjiu	278 (SSR02); 189 (SSR04); 192 (SSR07); 200 (SSR08); 184 (SSR09); 175 (SSR12); 159 (SSR14); 166 (SSR16)	8
Pudazi	195 (SSR07); 171 (SSR08); 179 (SSR12); 154 (SSR16)	4
Xuanliheshan	241 (SSR07); 167 (SSR08)	2
Zhengyan	393 (SSR01); 288 (SSR02); 212 (SSR04); 161 (SSR05); 234 (SSR08); 188 (SSR09); 159 (SSR10); 160 (SSR13)	8
Total		105

**Table 5:** The origins, regions, and codes of the 21 cultivars.

Cultivars	Origins	Regions	Codes of Cultivars	Autumn Leaf Color
Zilinglong	Chuzhou, Anhui	Anhui	20180091	Purple
Huangjinyi	Chuzhou, Anhui		20180391	Yellow
Hongfeicui	Guangde, Anhui		20150168	Red
Zimanao	Guangde, Anhui		20170053	Purple
Hongzijiaren	Qianxian, Anhui		20190340	Red
Zhaoxia	Qianxian, Anhui		20220211	Orange
Huangjinjia	Xuancheng, Anhui		20150167	Yellow
Huihuang	Dawu, Hubei	Hubei	20220215	Yellow
Canlan	Dawu, Hubei		20220214	Yellow
Haibinmenghuan	Dongtai, Jiangsu	Jiangsu	20180072	Red
Haibinfeihong	Lianyungang, Jiangsu		20180075	Purple
Qiuyan 01	Nanjing, Jiangsu		20160107	Red
Haibinzijing	Nanjing, Jiangsu		20180073	Purple
Feiyunzhaoshui	Nanjing, Jiangsu		20200119	Purple
Qiuhuang 01	Xinyi, Jiangsu		20160109	Yellow
Haibinwanxia	Xuzhou, Jiangsu		20180074	Purple
Ziyan	Zhenjiang, Jiangsu		20160108	Red
Puhongjiu	Pujiang, Zhejiang	Zhejiang	Zhe R-SV-SS-006-2018	Red
Pudazi	Pujiang, Zhejiang		20180397	Purple
Xuanliheshan	Pujiang, Zhejiang		20190339	Purple
Zhengyan	Suichang, Zhejiang		20220212	Red

**Table 6:** Information on the 16 polymorphic SSR markers.

Locus	Repeat Motif	Primer Sequence (5′~3′)	Fragment Size (bp)	Tm (°C)	Source
SSR01	(AG)10	AAACAAGTGAAGTGCCCAT	392	51	[1]
		TTAGCCCAGCCCATTATTA			
SSR02	(AAG)12	GGTTTCTTTTGCTCTCTTC	277	49	
		CCGGTTACTGCATTTCATA			
SSR03	(CA)11	CCAACAAGTTAGCATCACCT	115	58	[25]
		CAACAGAAGTTCCTCAATGT			
SSR04	(CT)15	CTCCAGCAGCTCTTCATCT	152	58	
		CGAACCAAGAATTAGGAAAAC			
SSR05	(AAG)10	GCCTTAAAGACATGGGATTC	126	58	
		CGATCCATTCTCTCTTGACA			
SSR06	(CTT)6	CTGATGGCAGTTCTTTGAGAT	203	58	
		GCCTGTTGTGGAATAGTGG			
SSR07	(AG)10	AACCCGTAAAGGGCTTGC	192	55	[27]
		CTGGTTCTCCTGGTTATCTATGC			
SSR08	(ATT)10	AAGGAATGGAGCGAAACGG	163	55	
		CCAATTGCGGCCATACTCG			
SSR09	(CTT)10	TCCGATCCAGTCCGTGTTG	184	55	
		GTGCGCGTGAGAGTGAATG			
SSR10	(CTT)9	TCTCTCCTTCGCTCAACGG	145	55	
		TCCGGGATCGGTGGAATTG			
SSR11	(CT)9	GTTTGTGAAGAGGGGTGAGC	179	55	
		AGTTGCTGAAATCCATACCATACC			
SSR12	(ATT)9	TGAACCTCGAACAAAAGTCAG	122	55	
		GTCAT(C/A)ATAACTTCGCGGG			
SSR13	(TAA)7	GTCAGCAGGGGAGAGCAAC	138	55	
		AATGGACAAAATGGCGCAC			
SSR14	(AG)16	AAGGAACCTGTTTGCTGGG	151	55	
		AAGTTCCGTTTCCACACGC			
SSR15	(CT)9GCC(TG)6	GTCAGTCGTCACCATCATCAG	202	55	
		CTACGACGACGCAACCAAC			
SSR16	(ATT)11	TCTTCGGGGAAACCGATCC	151	55	
		TGCTTTCAAAATGACACGGTTG			

## Data Availability

Data are contained within the article.

## Supplementary Materials

The following supporting information can be downloaded at: https://www.mdpi.com/article/10.3390/plants13131767/s1, Figure S1. Relationships between the number of clusters (K) and the corresponding ΔK.
