# Supplementary figures and images for: Genetic Analysis and Construction of a Fingerprint for Licensed Triadica sebifera Cultivars Using SSR Markers
Source: Plants (Basel). 2024 Jun 26;13(13):1767. doi: 10.3390/plants13131767 (PMC11244400; doi:10.3390/plants13131767)

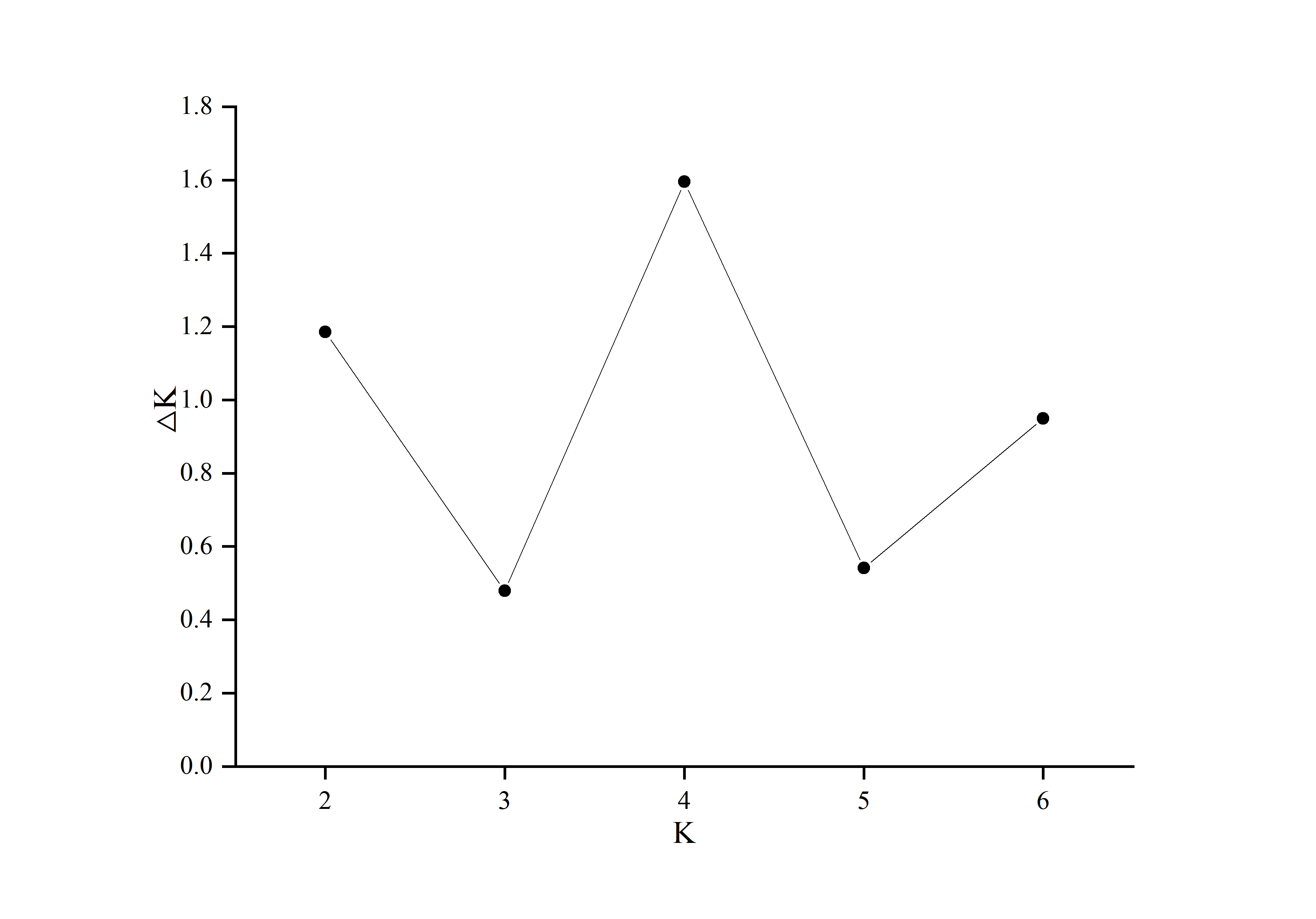

Supplement: Supplementary file 1 [file plants-13-01767-s001.zip › plants-3046171-supplementary.jpg]
